# The Effects of Signal Erosion and Core Genome Reduction on the Identification of Diagnostic Markers

**DOI:** 10.1128/mBio.00846-16

**Published:** 2016-09-20

**Authors:** Jason W. Sahl, Adam J. Vazquez, Carina M. Hall, Joseph D. Busch, Apichai Tuanyok, Mark Mayo, James M. Schupp, Madeline Lummis, Talima Pearson, Kenzie Shippy, Rebecca E. Colman, Christopher J. Allender, Vanessa Theobald, Derek S. Sarovich, Erin P. Price, Alex Hutcheson, Jonas Korlach, John J. LiPuma, Jason Ladner, Sean Lovett, Galina Koroleva, Gustavo Palacios, Direk Limmathurotsakul, Vanaporn Wuthiekanun, Gumphol Wongsuwan, Bart J. Currie, Paul Keim, David M. Wagner

**Affiliations:** a Center for Microbial Genetics and Genomics, Northern Arizona University, Flagstaff, Arizona, USA; b Translational Genomics Research Institute, Flagstaff, Arizona, USA; c Emerging Pathogens Institute, University of Florida, Gainesville, Florida, USA; d Global and Tropical Health Division, Menzies School of Health Research, Darwin, Northern Territory, Australia; e Pacific Biosciences, University of Michigan, Ann Arbor, Michigan, USA; f Division of Pediatric Infectious Diseases, University of Michigan, Ann Arbor, Michigan, USA; g Center for Genome Sciences, USAMRIID, Fort Detrick, Maryland, USA; h Mahidol-Oxford Tropical Medicine Research Unit, Mahidol University, Bangkok, Thailand; i Department of Tropical Hygiene, Faculty of Tropical Medicine, Mahidol University, Bangkok, Thailand

## Abstract

Whole-genome sequence (WGS) data are commonly used to design diagnostic targets for the identification of bacterial pathogens. To do this effectively, genomics databases must be comprehensive to identify the strict core genome that is specific to the target pathogen. As additional genomes are analyzed, the core genome size is reduced and there is erosion of the target-specific regions due to commonality with related species, potentially resulting in the identification of false positives and/or false negatives.

## IMPORTANCE

A comparative analysis of 1,130 *Burkholderia* genomes identified unique markers for many named species, including the human pathogens *B. pseudomallei* and *B. mallei*. Due to core genome reduction and signature erosion, only 38 targets specific to *B. pseudomallei*/mallei were identified. By using only public genomes, a larger number of markers were identified, due to undersampling, and this larger number represents the potential for false positives. This analysis has implications for the design of diagnostics for other species where the genomic space of the target and/or closely related species is not well defined.

## INTRODUCTION

Whole-genome sequence (WGS) data are routinely used to develop DNA-based diagnostics for rapid and accurate identification of clinical pathogens (1, 2). Validating the specificity of diagnostic targets ensures that assays do not produce false positives (identifying a nonpathogen as a pathogen) or false negatives (not identifying a pathogen that is actually present). To avoid false positives and negatives, DNA-based diagnostics must be conserved across the target species and absent from nontarget species.

Two critical issues arise during the process of identifying specific diagnostics from bacterial genomes. First, the number of genes in the core genome (i.e., genes present in every individual of a species) tends to become smaller as the number of sequenced genomes increases (3, 4). Certain pathogens (e.g., *Yersinia pestis*) propagate clonally, are highly homogeneous, and show little variation in core genome size with additional sampling (5). In this case, the core genome size is not expected to become drastically smaller as more genomes are analyzed. In contrast, the core genome size of *Burkholderia pseudomallei* becomes significantly smaller with each new genome added (6). A second issue arises from genomes of related species, or “near neighbors,” that share core genes with the target species. In a process of signature erosion, this genomic overlap often increases as near-neighbor genomes are added to the analysis, thus eroding the number of potential diagnostic targets. Unfortunately, near neighbors are often undersampled (or not sampled at all) during the search for diagnostic targets, which hinders efforts to identify species-specific targets.

*Burkholderia* represents a model genus for the demonstration of core genome reduction and signal erosion. The *Burkholderia* genus contains a diverse set of species, including plant pathogens (7) and human pathogens, such as *B. pseudomallei*, the causative agent of melioidosis (8), and *B. mallei*, the causative agent of glanders (9). The *pseudomallei* group includes *B. pseudomallei*, *B. mallei*, *B. oklahomensis*, *B. thailandensis*, and the newly described *B. humptydooensis* (10). The *B. cepacia* complex (Bcc) is a diverse group within *Burkholderia* that is associated with opportunistic infections and is comprised of at least 20 genomic species (11, 12). Most of the relationships between these species have been determined through gene marker analyses, such as the *recA* gene (13, 14) or multilocus sequence typing (15).

From a genomics perspective, *Burkholderia* whole-genome sequencing efforts have focused on *B. pseudomallei* (16) and *B. mallei* (17). Recent studies have begun to sequence other *Burkholderia* spp., including members of the Bcc (18). However, large-scale, whole-genome, phylogenetics-based studies that define the overall phylogenetic structure among *Burkholderia* species using high-resolution methods are currently lacking.

In this study, we extensively surveyed the environment in Australia, the United States, and Southeast Asia for *Burkholderia* spp. We sequenced a large collection of genomes to (i) explore the genomic diversity of *Burkholderia* spp. that grow on Ashdown’s agar, (ii) identify specific diagnostic markers for *B. pseudomallei* and *B. mallei*, and (iii) understand the sampling effects of core genome size reduction and signal erosion on the selection of highly specific diagnostic targets.

## RESULTS

### Whole-genome sequencing of *Burkholderia* spp.

In this study, we analyzed the whole-genome sequences of 829 *Burkholderia* spp. that grow on Ashdown’s agar (Table 1), a selective medium containing the aminoglycoside gentamicin. These isolates were collected from diverse geographic locations in the United States, Thailand, and Australia (see Table S1 in the supplemental material). To understand the effects of core genome reduction and signature erosion on the identification of highly specific diagnostic targets, the genomes of 256 diverse *B. pseudomallei*/*mallei* strains were sequenced, assembled, and deposited in public databases (see Table S1); these genomes were combined with 160 *B. pseudomallei*/*mallei* genome assemblies already in public databases. Most of the genomes (*n* = 779) in this study were sequenced on the Illumina platform, with 50 genomes also sequenced on the PacBio platform, which generated highly contiguous and often finished assemblies (see Table S1).

**TABLE 1:** Summary of new genomes sequenced as part of this study

Clade	No. of genomes
*B. anthina*	8
*B. cenocepacia* 1	1
*B. cenocepacia* 2	4
*B. cepacia*	78
*B. diffusa*	12
*B. gladioli*	1
*B. humptydooensis*	5
*B. lata*	2
*B. latens*	2
*B. metallica*	1
*B. multivorans*	14
*B. oklahomensis*	2
Putative species 1	3
Putative species 2	4
Putative species 3	10
Putative species 4	7
Putative species 5	8
*B. pseudomallei*	256
*B. pseudomultivorans*	9
*B. pyrrocinia*	1
*B. seminalis*	2
*B. stagnalis*	67
*B. thailandensis*	8
*B. territorii*	33
*B. ubonensis*	254
*B. vietnamiensis*	37
Total	829

### Core genome SNP phylogeny.

**FIG 1:**
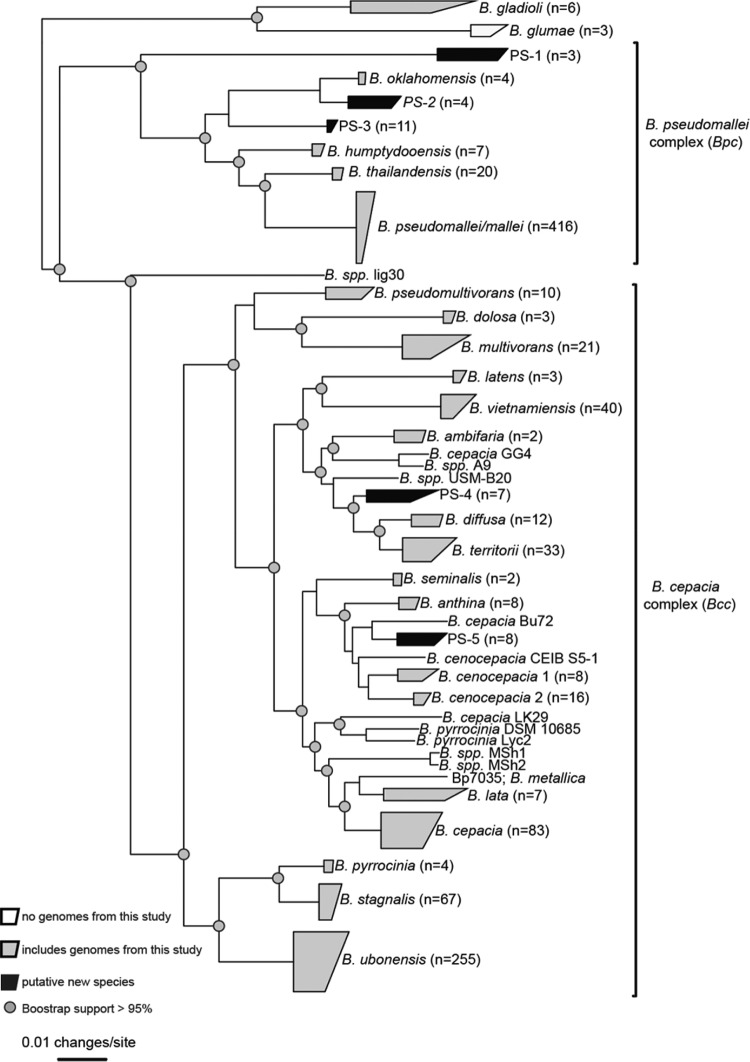
A core genome single-nucleotide-polymorphism (SNP) phylogeny of *Burkholderia* genomes. All SNPs were identified by aligning genome assemblies against the finished genome of *B. pseudomallei* K96243 (19) with NUCmer (20) and processed with the Northern Arizona SNP Pipeline (http://tgennorth.github.io/NASPtgennorth.github.io/NASP) (30). A maximum-likelihood phylogeny was inferred on the concatenated SNP alignment with RAxML version 8 (31) with 100 bootstrap replicates. Clades were collapsed with ARB (41). Putative novel species are named with PS (putative species) and the clade number.

To understand the phylogenetic structure of the *Burkholderia* genus, genomes sequenced in this study, as well as GenBank reference genomes (see Table S2 in the supplemental material), were aligned against *B. pseudomallei* strain K96243 (19) with NUCmer (20) and single-nucleotide polymorphisms (SNPs) were identified with NASP. The maximum-likelihood phylogeny inferred from core, orthologous SNPs (*n* = 105,877) demonstrated that all genomes sequenced in this study, with the exception of 1 *B. gladioli* genome, grouped in either the *Burkholderia cepacia* complex (Bcc) or the *B. pseudomallei* group (Fig. 1). Based on the monophyletic nature and complexity of the latter clade, we propose to name it the *B. pseudomallei* complex (Bpc). Multiple additional *Burkholderia* genomes from GenBank were analyzed and were found to be more distantly related to these two groups. As such, they were not examined in detail in this study but were included for marker-screening purposes (see “Paraburkholderia” genomes in Table S2). Our clade naming scheme is consistent with a recently published taxonomic scheme for *Burkholderia* (21).

The work performed in this study greatly expands the known genomic diversity of *Burkholderia*. For example, at the outset of this study, only two *B. ubonensis* genome assemblies were available in GenBank. This is likely due to the fact that most genome sequencing has focused on clinically relevant organisms, whereas we sampled both environmental and clinical isolates. This study adds the genomes of 254 *B. ubonensis* isolates, including three finished genomes (3 contigs) and three nearly finished genomes (4 to 5 contigs) (see Table S1 in the supplemental material). All of these genomes are publicly available and will help provide phylogenetic context for additional *Burkholderia* genomes that are sequenced, including from clinical isolates. We have also generated the first whole-genome sequences for other recently described species, such as *B. stagnalis* and *B. territorii* (12), including completed genomes, which will provide data for additional comparative studies.

### Comparative genomics.

**FIG 2:**
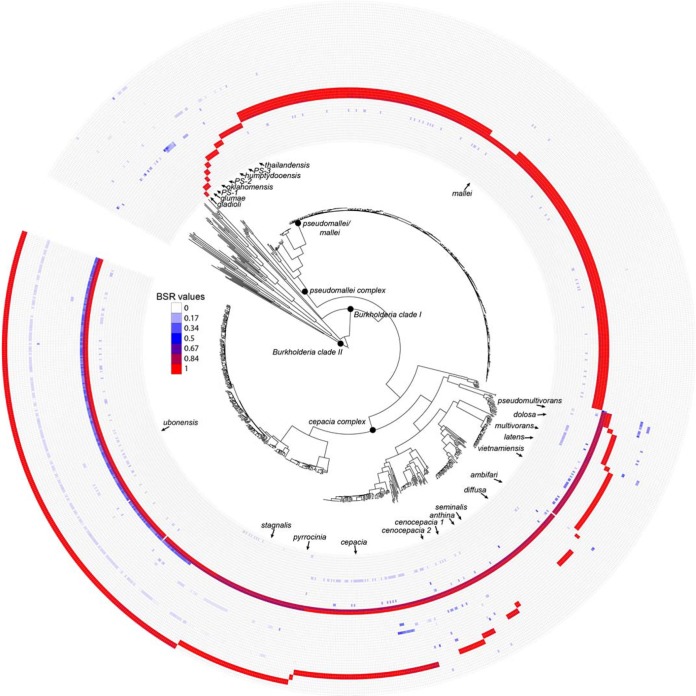
A core genome single-nucleotide-polymorphism (SNP) phylogeny associated with a heat map of markers unique to specific clades. The core genome phylogeny was inferred with RAxML (31) on a concatenated SNP alignment produced by aligning 1,130 genomes against the finished genome of *B. pseudomallei* K96243 (19), using NUCmer (20) in conjunction with NASP (http://tgennorth.github.io/NASPtgennorth.github.io/NASP). Coding regions unique to specific clades were aligned against all genomes with LS-BSR (22), and the heat map was visualized with the Interactive Tree of Life (42). The heat map demonstrates the distribution of identified markers against all genomes screened in this study.

Based on the topology of the core genome phylogeny (Fig. 1), pangenome statistics were calculated for each major clade (see Table 2 in the supplemental material) using the large-scale BLAST score ratio (LS-BSR) pipeline (22). The core genome of each primary clade was aligned against all surveyed genomes (*n* = 1,130) to identify species- or clade-specific markers. A marker was determined to be clade specific if it had a BSR of >0.8 in all target genomes and <0.4 in all nontarget genomes; although this definition is very conservative, it was used to identify discriminatory markers, regardless of genome assembly quality. The results demonstrate that species-specific markers were identified for most of the major clades (Table 2); a multi-FASTA file of all species-specific coding regions is publically available (https://gist.github.com/jasonsahl/3e4132ca1d09b717fcc2). A screen of these species-specific markers against all genomes was visualized to demonstrate their specificity to each targeted clade (Fig. 2). The stability of markers from clades with a limited number of representatives is unknown and will need to be validated with additional sequencing. Markers also were identified for the *B. cepacia* complex (Bcc) and the *B. pseudomallei* complex (Bpc), which can help to verify results obtained through diagnostic sequencing efforts.

**TABLE 2:** Core genome statistics

Species/clade	Core genome size (CDSs)	No. of:	
		Genomes	Species-/clade-specific markers
*ambifaria*	5,408	2	71
*anthina*	5,507	8	13
*cenocepacia* 1	3,823	8	8
*cenocepacia* 2	5,076	16	22
*cepacia*	4,415	83	7
*diffusa*	4,566	12	7
*dolosa*	5,451	3	436
*gladioli*	4,898	6	833
*glumae*	3,253	3	264
*humptydooensis*	5,115	7	157
*lata*	4,214	7	0
*latens*	5,348	3	105
*multivorans*	4,001	21	53
*oklahomensis*	5,681	4	141
PS-1	3,693	3	504
PS-2	4,231	4	23
PS-3	5,047	11	195
PS-4	4,366	7	0
PS-5	4,978	8	0
*pseudomallei*	2,339	392	22
*pseudomallei/mallei*	1,690	416	38
*pseudomultivorans*	4,549	10	62
*pyrrocinia*	6,397	4	153
*seminalis*	6,533	2	90
*stagnalis*	4,835	67	54
*thailandensis*	4,447	20	116
*territorii*	4,399	33	0
*ubonensis*	3,128	255	40
*vietnamiensis*	3,803	40	71

### Putative new species.

Based on the phylogeny (Fig. 1), five divergent clades were identified that may represent novel species (PS-1 through PS-5). We have generated completed or nearly completed genomes for at least one isolate from each of these clades (Table 1). A BLASTN alignment of the extracted *recA* sequences against the GenBank nucleotide database failed to identify a close match to a named species for any of these clades. To demonstrate the differences between genomes in these putative species, one representative was compared against a genome of the nearest species, based on the closest patristic distance, or tree path distance, to the nearest monophyletic clade in the global phylogeny (Fig. 1). For each pairwise comparison, the average nucleotide identity (ANI) and DNA-DNA hybridization (DDH) values were calculated and tabulated (Table 3). The results demonstrate that many of the clades have ANI values of <95% compared to the nearest reference genome based on its position in the phylogeny. Putative species 2 (PS-2), which is most closely related to *B. oklahomensis*, demonstrated ANI values on the border of the species threshold compared to *B. oklahomensis* genomes. All of the genomes from PS-2 have been isolated from Australia, whereas all *B. oklahomensis* genomes, including the two publicly available genomes, have been isolated from the United States (see Table S1 in the supplemental material). This physical separation, combined with the borderline ANI values, may argue for separate species, but biochemical testing is required to bolster this separation and is currently ongoing.

**TABLE 3:** Average nucleotide identity and DNA-DNA hybridization values between representatives of putative novel species and representatives of established clades^a^

Genome	Clade	Nearest genome	ANIm (%)	ANIb (%)	DDH range (%)
MSMB175	Putative species 1	*B. gladioli* BSR3	85.5	79.8	18.7–23.7
BDU8	Putative species 2	*B. oklahomensis* C6786	94.9	94.8	59.3–75.8
MSMB0852	Putative species 3	*Burkholderia* sp. strain MSMB43	92.4	91.1	44.5–52.7
MSMB0856	Putative species 4	*B. pyrrocinia* lyc 2	91.2	89.8	44.9–60.8
NRF60-BP8	Putative species 5	*B. cenocepacia* KC-01	94.1	93.5	54.5–56.9

a ANI, average nucleotide identity; ANIm, uses NUCmer alignments; ANIb, uses BLASTN alignments; DDH, DNA-DNA hybridization.

### Core genome size reduction with additional sampling and signal erosion with the inclusion of near-neighbor genomes.

**FIG 3:**
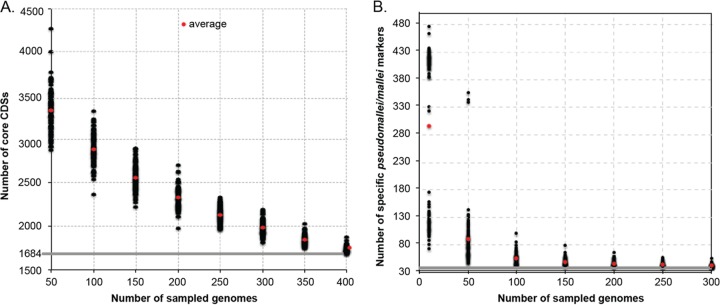
(A) Core genome reduction in *Burkholderia pseudomallei*/mallei. The core genome was calculated with the LS-BSR pipeline (22) on 416 genomes. For subsampling, genomes were randomly selected at different depths and the number of coding regions (CDSs) with a BLAST score ratio (BSR) (39) of >0.8 in all genomes was calculated and plotted. For each subsampling level, 100 iterations were performed. The mean value at each level is shown in red, and each replicate is shown in black. (B) The effect of signature erosion on the design of *B. pseudomallei*/mallei diagnostic markers. Genomes outside the *B. pseudomallei*/mallei clade (*n* = 714) were randomly selected at different depths. The core genome of 416 *B. pseudomallei*/mallei genomes was screened against non-*pseudomallei*/mallei genomes with LS-BSR (22), and the number of markers with a BSR of <0.4 in non-*pseudomallei*/mallei genomes was calculated and plotted. One hundred independent replicates were processed at each sampling depth. The mean value at each level is shown in red, and each replicate is shown in black.

In bacteria with highly plastic genomes, the inclusion of additional isolates can cause the core genome size to decrease (3). To demonstrate this effect in *Burkholderia*, we calculated pangenome statistics on 416 *B. pseudomallei*/*mallei* genomes. The results demonstrated that as additional genomes were added to the analysis, the core genome size reduced to 1,684 coding DNA sequences (CDSs); annotation of these CDSs is provided in Table S3 in the supplemental material. This analysis included genomes from *B. mallei*, which has undergone significant evolutionary decay (9), and isolates from a chronic *B. pseudomallei* infection that have also undergone substantial genome reduction over time due to long-term host adaptation (23). By inclusion of a diverse set of genomes, the minimum set of genes required by all *B. pseudomallei*/mallei bacteria could be identified. From randomly subsampling the 416 genomes at different genome levels, the sampling effect on the core genome size was visualized (Fig. 3A).

In addition to core genome size reduction, the effect of including additional near-neighbor genomes on accurate diagnostics was also investigated. The core genome from all *B. pseudomallei*/mallei genomes was aligned using LS-BSR against a randomly selected subset of near-neighbor genomes ranging from 10 to 300 genomes, with each iteration performed 100 times. By the time 300 near-neighbor genomes were randomly selected, the number of *B. pseudomallei*/mallei markers converged on the same number that was obtained using the entire set of 714 near-neighbor genomes (Fig. 3B). This result demonstrates that a significant number of near-neighbor genomes must be sequenced in order to identify a set of molecular markers that are highly discriminatory for a given clade.

When we considered the 416 *B. pseudomallei*/mallei genomes, a surprisingly small number of unique markers (*n* = 38) were identified (see Table S3 in the supplemental material). Of these markers, one in particular (BPSS0060; encodes a hypothetical protein) only contained 3 polymorphisms across all of the diverse *B. pseudomallei*/mallei isolates in our study. This gene represents a highly specific diagnostic target that is under low selection for mutation. If only *B. pseudomallei* was considered, 22 conserved markers were identified (see Table S4) in *B. pseudomallei* that were missing from *B. mallei* and all other *Burkholderia* genomes considered (Fig. 2).

If we only consider the publicly available genomes used in this study (*n* = 298), the core genome size for *B. pseudomallei*/mallei is 2,570 CDSs. When this core genome was screened against other *Burkholderia* near-neighbor genomes available in GenBank (*n* = 141), 63 markers were identified that were unique to *B. pseudomallei*/mallei. In contrast, if near-neighbor genomes sequenced in the study were also included (*n* = 573), 51 markers were identified. By not including additional nontarget reference genomes, 13 of these markers would represent false positives in screening studies. If only target genomes in GenBank were considered, 25 false positives would be identified, demonstrating the need to include large numbers of target and nontarget genomes.

## DISCUSSION

Accurate design of highly specific diagnostics is important for the detection of dangerous human pathogens in both environmental and clinical settings. Timely pathogen identification directly from clinical specimens could inform the early treatment of potentially deadly infections. However, as our study demonstrates, the genomic targets of molecular assays need to first be thoroughly validated to avoid false positives and false negatives, which can potentially confound diagnostic tests and delay appropriate patient treatment. In this study, we highlight the importance of exploring the strict core genome size and signature erosion before designing diagnostic PCR targets. Our genome-based approach is applicable for other researchers who wish to develop diagnostic assays for other pathogens.

The effects of signature erosion and core genome size reduction were highlighted in the genus *Burkholderia*. To characterize the genomic space within *B. pseudomallei*/mallei, as well as in closely related genomes, we sequenced 829 *Burkholderia* genomes from diverse locations. A large-scale comparative genomics analysis of these genomes demonstrated that specific molecular markers were identified for many of the major *Burkholderia* clades identified from the core genome SNP phylogeny (Fig. 2). These unique coding regions were likely acquired horizontally, based on the lack of homology of these regions to other lineages within the genus (Fig. 2). To demonstrate the need to sequence a large collection of genomes to identify specific diagnostic targets, a core genome reduction analysis was performed (Fig. 3A). This analysis demonstrated that sequencing additional genomes causes the core genome size to decline. This analysis was performed by including a large number of draft genome assemblies, which may cause genomic elements to be truncated, based on unresolvable repeats, or missing altogether, based on either insufficient coverage or assembly algorithms that remove either short contigs or regions of anomalous coverage. Based on the genome panel used in this analysis, including a large and diverse set of isolates is important to avoid selecting potential diagnostic targets that are susceptible to false-negative results when screening either clinical or environmental samples. If only genomes in public databases were selected, multiple markers would be identified that represent potential false negatives.

The other important factor to consider when designing diagnostic markers is the effect of signature erosion that can be introduced due to the inclusion of close relatives to the clade of interest. If only genomes available in GenBank were included in the analysis, 63 markers were identified that appeared to be specific to *B. pseudomallei*/mallei. However, if all non-*pseudomallei*/mallei genomes were included in the analysis, only 51 *B. pseudomallei*/mallei markers were identified, demonstrating the impact of including a comprehensive set of genomes outside the targeted species or clade. If all genomes from our study were included, only 38 *B. pseudomallei*/mallei-specific markers were identified, which demonstrates the need to include diverse genomes both from the targeted clade and from clades closely related to the targeted clade.

This study both expands the known genomic diversity of the *Burkholderia* genus and provides a framework for using genomic data to design highly specific diagnostic targets. For some species, near-neighbor genomes are not available or are difficult to isolate, which complicates the identification of these targets and highlights the need for continued genome sequencing. The reported sampling effects on strict core genome size and signature erosion must be considered when interpreting surveillance results for human pathogens.

## MATERIALS AND METHODS

### Isolate collection, DNA extraction, genome sequencing, assembly.

*Burkholderia* isolates were collected from diverse global locations, with a focus on regions where *B. pseudomallei* is highly endemic, including northern Australia and northeastern Thailand (see Table S1 in the supplemental material). Isolates were collected by the Menzies School of Health and Research, Northern Arizona University, the University of Michigan, the James Cook University, Mahidol University, and the U.S. Army Medical Research Unit (USAMRU). All final culture and DNA extraction procedures were performed at Northern Arizona University, and whole-genome sequencing (WGS) was performed at the Translational Genomics Research Institute (TGen), using Illumina, and the U.S. Army Medical Research Institute of Infectious Diseases (USAMRIID), using PacBio.

Isolates initially grown on Ashdown’s agar were streaked from a single purified colony to form a lawn and then stored at −80°C in Luria Bertani (LB) broth with 20% glycerol. Cultures were grown on LB agar plates and incubated at 37°C for 24 to 48 h. High-molecular-weight DNA was extracted using the Qiagen DNeasy blood and tissue kit (catalog no. 69504; Valencia, CA) for whole-genome sequencing on the Illumina (Illumina, Inc., San Diego, CA) and Pacific Biosciences (Menlo Park, CA) platforms. Using approximately 2.7 µg of genomic DNA (gDNA), libraries were prepared for Illumina whole-genome sequencing as previously described (24).

DNA was sequenced on multiple platforms, including Illumina HiSeq 2000, Illumina MiSeq, and PacBio. Raw Illumina reads were assembled with SPAdes version 3.5.0 (25) in conjunction with a pipeline developed to identify sequence contamination between multiplexed samples (https://github.com/jasonsahl/UGAP). Contigs that either showed an anomalously low depth of coverage compared to other contigs from the same assembly or aligned to other organisms multiplexed in the same lane were manually removed. Genome assembly information is shown in Table S1 in the supplemental material.

For PacBio assemblies, genomic DNA was sheared to an average size of 20 kb using g-TUBEs (Covaris, Inc.). After DNA damage repair and end repair, hairpin adapters were ligated to form a SMRTbell template. ExoIII and ExoVII treatment was used to remove failed ligation products. Size selection was performed on the Blue Pippin system (Sage Sciences) using a 0.75% dye-free agarose gel cassette, marker S1, and Hi-Pass protocol; the low cut was set on 4,000 bp. The final library assessment was obtained with the Qubit double-stranded DNA (dsDNA) BR assay and Agilent 2100 Bioanalyzer DNA 12000 chip analyses. Annealing of the sequencing primer and binding polymerase P4 to the SMRTbell template was performed according to the PacBio calculator. The polymerase-template complexes were bound to MagBeads, loaded onto SMRTcells at a final concentration of 180 pM, and sequenced with 180-min movies on the PacBio RS II instrument.

PacBio sequences were assembled *de novo* using the Hierarchical Genome Assembly Process (HGAP) (26). Draft assemblies were checked for overlapping ends using Gepard (27) and BLAST (28). Overlapping ends are typical of long-read assemblies of circular chromosomes. Redundant end sequences were trimmed to one copy, and the genome was rotated to create a new breakpoint. Reads were then realigned to the trimmed and shifted draft assembly for correction using the Quiver algorithm. Contigs that did not have identifiable homologous ends were corrected using Quiver without further processing.

### Species identification using core genome SNP phylogeny, ANI, and DDH calculation.

To model the evolutionary relationships between *Burkholderia* spp., a set of reference genomes (see Table S2 in the supplemental material) was downloaded from GenBank (29) and combined with the genomes sequenced in this study. For a number of these genomes, only raw reads were available, which were assembled for use in the comparative analyses described below. All genomes were aligned against the reference genome of *B. pseudomallei* K96243 (19) using NUCmer (20). Regions that aligned more than once by a reference self-alignment (i.e., duplicated regions) were removed from downstream analyses. All SNP-based methods were wrapped by the Northern Arizona SNP pipeline (NASP) (http://tgennorth.github.io/NASPtgennorth.github.io/NASP/) (30). Orthologous SNPs conserved in all genomes were concatenated, and a maximum-likelihood phylogeny was inferred with RAxML version 8 (31) using the ASC_GTRGAMMA substitution model and Lewis correction (32).

For determining species differences, the average nucleotide identity (ANI) was calculated with default values in JSpecies (33). JSpecies calculates ANIb, which uses BLASTN alignments (28), or ANIm, which uses NUCmer alignments. The average values were reported over the entire length of all alignments. To find the nearest neighbor to which to query target genomes, the closest patristic distances were chosen, as calculated by DendroPy (34). Digital DNA-DNA hybridization (DDH) values were calculated with a Web service (http://ggdc.dsmz.de) (35), and the range of reported values is presented.

### Identifying *B. pseudomallei* and *B. mallei* markers for diagnostics using comparative genomics and pangenome analysis.

Coding DNA sequences (CDSs) were identified for each species with Prodigal (36) and were dereplicated with USEARCH (37). Each representative CDS was then aligned against each genome with BLAT (38), and the BLAST score ratio (BSR) (39) was calculated; these methods were all wrapped by the large-scale BLAST score ratio (LS-BSR) pipeline (22). LS-BSR was performed for each species, and the number of core CDSs (BSR of >0.8 in all genomes) in each group was calculated; a BSR of 0.8 is roughly equivalent to 80% protein identity over 100% of the length of the protein (3). These core CDSs from a given species or clade were then screened against all other genomes, and those genes with a BSR of <0.4 in all other species were identified as suitable species diagnostic markers.

The pangenome was calculated for each clade using LS-BSR in conjunction with BLAT. A CDS was determined to belong to the core genome if it had a BSR of >0.8 in all genomes queried for a given species or clade of interest. Each core CDS was then screened against all genomes in the analysis with LS-BSR. A CDS was determined to be species specific if it was in the core genome of the species or clade of interest and missing or highly divergent (BSR of <0.4) in all other *Burkholderia* genomes.

### Core genome size reduction and signal erosion.

To understand the sampling effect on the core genome size in *B. pseudomallei*/mallei, a set of 416 *B. pseudomallei*/mallei genomes was sampled without replacement from 1 to 400, with 100 iterations at each level. From each subsampling, a set number of genomes were randomly selected with a Python script (https://gist.github.com/990d2c56c23bb5c2909d.git), and the core genome (CDSs with a BSR of >0.8 in all genomes) was calculated and plotted. *B. pseudomallei* and *B. mallei* were treated as a single species for this and many of the subsequent analyses, as *B. mallei* is recognized as an equine-adapted clone within *B. pseudomallei* (40).

To understand the erosion of *B. pseudomallei*/mallei-specific targets with the inclusion of sequences from other *Burkholderia* spp., the core genome (*n* = 1,684 CDSs) from a set of 416 *B. pseudomallei*/mallei genomes was used. All *Burkholderia* near-neighbor genomes (*n* = 714) were then randomly sampled without replacement at different levels from 1 to 300. The *B. pseudomallei*/mallei core genome was then aligned against these near-neighbor genomes to identify core regions present in other *Burkholderia* species, and the number of CDSs with a BSR of <0.4 in all near-neighbor genomes, indicating missing genes, was calculated and plotted.

### Accession numbers.

Sequence data were submitted to the Sequence Read Archive for each isolate. Furthermore, genome assemblies for all isolates were submitted to NCBI. Individual accession numbers are shown in Table S1 in the supplemental material, and all data are deposited under BioProject accession numbers PRJNA285704 and PRJNA279182.

## SUPPLEMENTAL MATERIAL

Table S1  Details of genomes sequenced in the current study. Table S1, XLSX file, 0.1 MB

Table S2  Accession information for genomes screened in this study. Table S2, XLSX file, 0.1 MB

Table S3  Locus tag information for the *B. pseudomallei*/mallei core genome. Table S3, XLSX file, 0.1 MB

Table S4  Core genome regions across all *B. pseudomallei* genomes. Table S4, XLSX file, 0.1 MB
